# Trends in occupational diseases in the Italian agricultural sector, 2004–2017

**DOI:** 10.1136/oemed-2019-106168

**Published:** 2020-01-29

**Authors:** Henk F van der Molen, Chiara Marsili, Andrea Vitali, Claudio Colosio

**Affiliations:** 1 Amsterdam UMC, University of Amsterdam, Department: Coronel Institute of Occupational Health, Netherlands Center for Occupational Diseases, Amsterdam Public Health research institute, Amsterdam, The Netherlands; 2 Occupational Health Unit and International Centre for Rural Health, ASST of the Saints Paolo and Carlo, Milano, Italy; 3 University of Milan, Department of Health Sciences, Occupational Health Unit and International Centre for Rural Health, ASST of the Saints Paolo and Carlo, Milano, Italy

**Keywords:** epidemiology, occupational diseases, Agriculture

## Abstract

**Objective:**

To estimate the incidence of, trends in and effect of change in reporting rules on occupational diseases (ODs) in the Italian agricultural sector.

**Methods:**

Over a 14-year period (2004–2017), ODs among Italian agricultural workers were diagnosed by physicians and reported to the National Institute for Insurance against Workplace Accidents and Occupational Diseases. OD was defined as a disease with a specific clinical diagnosis (International Classification of Diseases) and was predominantly caused by work-related factors. Trends in incidence and effects of changed eligibility criteria for reporting occupational musculoskeletal disorders (MSDs) and noise-induced hearing loss (NIHL) were estimated using a Poisson regression model.

**Results:**

In 2017, the incidence of all ODs was 1295 per 100 000 agricultural workers. MSDs (961 per 100 000 workers) were the most frequently occurring ODs. MSDs and NIHL showed statistically significant increasing time trends, 26% and 7% annual increase, respectively, during the 2004–2017 period. There was no statistically significant change in the incidence of occupational respiratory, skin and cancer diseases during the 14-year period. After changes in reporting rules, the incidence of MSDs showed an immediate increased effect, with an incidence rate ratio (IRR) of 2.9 (95% CI 2.65 to 3.14) and a significant annual decreasing trend of −9% (95% CI −6% to −12%) over the years after the changed reporting rules (from 2008 to 2017), and an immediate effect on NIHL with an IRR of 1.3 (95% CI 1.13 to 1.53).

**Conclusion:**

In total, 1.3% of the Italian agricultural workers were diagnosed in 2017 as having an OD. Over a 14-year period, the annual incidence of ODs showed a considerable increasing trend consistent with changed eligibility reporting criteria for occupational MSDs and to a lesser extent for NIHL.

**Key messages**

**What is already known about this subject?**

- Agriculture is considered one of the most hazardous production sectors, with a very high burden of occupational accidents and diseases.

**What are the new findings?**

- 1.3% of workers in the Italian agricultural sector were diagnosed with an occupational disease in 2017.
- Over a 14-year period, the annual incidence of reported occupational diseases showed a statistically significant increase, mainly due to changed eligibility criteria.

**How might this impact on policy or clinical practice in the foreseeable future?**

- The increase in incidence of occupational diseases is not indicative of a worsening working condition but consistent with changed eligibility criteria for reporting of occupational diseases.

## Introduction

More than one-third of the global workforce is employed in the agricultural sector, engaged in activities both indoors and outdoors, with different levels of mechanisation. Agriculture is considered one of the most hazardous production sectors, with a very high burden of occupational accidents and diseases.^1^ Agricultural workers are exposed to machineries and tools, vehicles, various animals and plants, noise and vibration, slips, trips and falls, and physically demanding work.^1 2^ Other typical risks are exposure to organic and non-organic dusts, chemicals and biological agents, and exposure to extreme temperatures.^1 3^ This diverse and cumulative exposure increases the risk of occupational diseases (ODs) in the agricultural sector; however, epidemiological data are scarce and significant under-reporting is probably present.^3^ Reasons for under-reporting are the lack of occupational health structures in rural areas, the lack of legislation addressing workers’ health surveillance for family and small-sized enterprises and the self-employed, and the lack of specific legislation addressing ODs.^3 4^ Under Italian legislation, all ODs and exposure conditions to be mandatorily covered by insurance are listed in a list annexed to an Italian law; reporting ODs is the duty of any physician, and is in particular the first to make the diagnosis independently from their specific specialisation. In 2008, new legislation was adopted and eligibility criteria for reporting ODs were changed, adding musculoskeletal disorders (MSDs) with International Classification of Diseases (ICD-10) coding to the Italian list and adding exposure criteria (daily or weekly exposure to noise at levels exceeding 80 dB(A)) for noise-induced hearing loss (NIHL). Knowledge of the OD burden in the agricultural sector is a prerequisite to deciding the needs and priorities for preventive activities and intervention.^3^ Measuring the incidence and trends in ODs may increase the potential to develop, implement and evaluate individual and sectorial interventions aimed at reducing OD-related risk factors. It is also interesting to consider the reporting behaviour of physicians in the light of the changes in 2008 to the eligibility criteria for reporting ODs.

The objectives of this study are to determine (1) the incidence of reported ODs in 2017; (2) the trends in OD incidence rate ratio (IRR) over a 14-year period (2004–2017); and (3) an estimate of the effect of the changes in 2008 to the eligibility criteria for reporting occupational MSDs and NIHL in the Italian agricultural sector. It was hypothesised that only ODs for which eligibility criteria for reporting were changed, that is, MSDs and NIHL, would be expected to change in incidence rate.

## Methods

### Study design and procedures

As part of a dynamic prospective cohort over a 14-year period (2004–2017), all ODs reported to Italy’s National Institute for Insurance against Workplace Accidents and Occupational Diseases (INAIL) in the national agricultural sector were considered. Data refer to ODs reported, not acknowledged and compensated. The process of compensation is in some cases quite lengthy (eg, compensation can be denied, a worker can claim a revision), but occupational physicians (OPs) are also unlikely to report an OD that does not have a good chance of compensation.

OD was defined as a disease with a specific clinical diagnosis (ICD-10 classification) and was predominantly caused by work-related factors, and was listed in the Italian List of Occupational Disease to be mandatorily covered by insurance.^5^ The list is based on specific diseases, identified with their ICD-10 codes, linked with specific circumstances of exposure. When a diagnosis is made, and the disease together with one of the indicated circumstances of exposure is present, the disease is automatically compensated. If a disease is suspected to be occupational but is not on the list, and/or the list does not include the circumstances of exposure of the specific case, a claim of OD can be made; however, the claimant is expected to be able to demonstrate the occupational origin of the disease.

Diagnosis can be made based on clinical evidence accompanied, when necessary, by selected clinical tests (eg, allergic prick testing, measuring specific IgE antibodies to allergens, pulmonary function assessment, hearing capacity assessment). Exposure is usually indicated as ‘present’ or ‘absent’ based on the presence of the circumstances of exposure on the list. Environmental data can only be requested in case of doubt. Reporters are all medical doctors who made the first diagnosis, mainly general practitioners, hospital specialists of various disciplines and occupational physicians working in the locality, at the enterprise level, as well as in the occupational health units of hospitals. The list was updated in 2008 with the inclusion of new diseases and of new circumstances of exposure for some diseases already listed.

Incidence was determined for the following five groups of diseases present in the Italian list: NIHL: H833 (new circumstances of exposure added in 2008); MSD: G56.0, M19.2, M23.3, M51.2, M65.4, M65.8, M70.2, M70.4, M75, M75.1, M75.2, M75.3, M75.5, M76.8, M77.0, M77.1 (mostly added in 2008); cancers: C11, C22.3, C30, C31, C34, C44, C45.0, C45.1. C45.2, C45.7, C67, C82-C96 (unchanged in 2008); skin diseases: L23, L24, L24.1, L57, L57.0, L70.8 (unchanged in 2008); and respiratory diseases: I70, J40, J44 J45.0, J.62.0, J63.0, J63.8, J66.0, J67, J68.1, J68.4 (unchanged in 2008).

### Analysis

Annual incidence was determined by dividing the number of reported ODs per year by the total number of agricultural workers that year (provided by INAIL). INAIL insurance is mandatory for enterprises whose main source of income is agriculture (certified by tax paid), and for other companies this insurance is voluntary; the total number of insured is around 500 000. In total, about 95% are small-sized and medium-sized and family enterprises.

To estimate trends in incidence, annual case counts were analysed using a Poisson regression model. Time (year) was treated as a continuous variable, and population estimates, as natural logarithms of the annual number of workers, were included in the regression model as an ‘offset’. A modification of the parameterisation of Ramsay *et al* 6 was used to estimate the effect of the changed eligibility criteria for reporting occupational MSDs and NIHL in 2008, while taking into account secular time trends. Details of the model specification are as follows: Y=ß0+ß1time+ß2 (time-p) I(time >p)+ß3 I(time >p). For time=1,…, T, where p is the time of the start of the changed eligibility criteria, I(time ≥p) is a function that takes the value of 1 if time is p or later and 0 otherwise. The parameters ß have the following interpretations: ß1 is the time trend before the changed eligibility criteria for reporting ODs (2004–2007), ß2 is the difference between the time trends before and after the changed eligibility criteria for reporting ODs (2004–2007 vs 2008–2014), and ß3 is the immediate change in level of IRR at the start of the changed eligibility criteria for reporting ODs in 2008, that is, the difference between the IRR in 2008 and that predicted by the preintervention time trend. All analyses were performed with Stata V.15.

## Results

**Figure 1:**
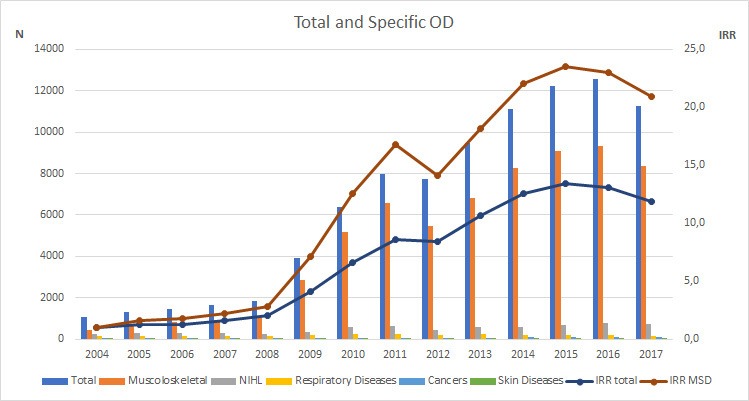
Absolute number (N) and incidence rate ratio (IRR) of reported occupational diseases (ODs) in the Italian agricultural sector in 2004–2017. MSD, musculoskeletal disorder; NIHL, noise-induced hearing loss.

In total, 90 040 ODs among Italian agricultural workers over a 14-year period were reported (see figure 1). The annual population of agricultural workers varied from 990 000 in 2004, to 812 000 in 2014, to 872 000 in 2017, with an average of 883 867 workers (SD: 54 588).

Table 1 shows the total annual incidence of ODs to be 1295 per 100 000 workers in the Italian agricultural sector in 2017; 74% of the diagnoses were MSDs. The highest incidence of ODs was reported in relation to MSDs (961 per 100 000 workers). The incidence of occupational NIHL, respiratory, cancer and skin diseases was, respectively, 84, 19, 11 and 4 per 100 000 workers.

**Table 1:** Incidence of occupational diseases in the Italian agricultural sector

Diagnosis (ICD-10)	Incidence in 2017 per 100 000	Trends over time, 2004–2017 period 14 years		
		Occupational diseases (n)	IRR	95% CI
Total	1295	90 040	1.16	1.14 to 1.19
Musculoskeletal disorders	961	65 935	1.26	1.22 to 1.31
Lumbar disc herniation	413			
Shoulder disorders	325			
Noise-induced hearing loss	84	6737	1.07	1.01 to 1.12
Skin diseases	4	490	1.17	0.96 to 1.42
Respiratory diseases	19	2752	1.01	0.94 to 1.08
Asthma	9			
Cancer	11	802	1.15	0.97 to 1.37

IRR is the annual change in incidence from 2004 to 2017 assuming a linear trend.

ICD-10, International Classification of Diseases; IRR, incidence rate ratio.

**Figure 2:**
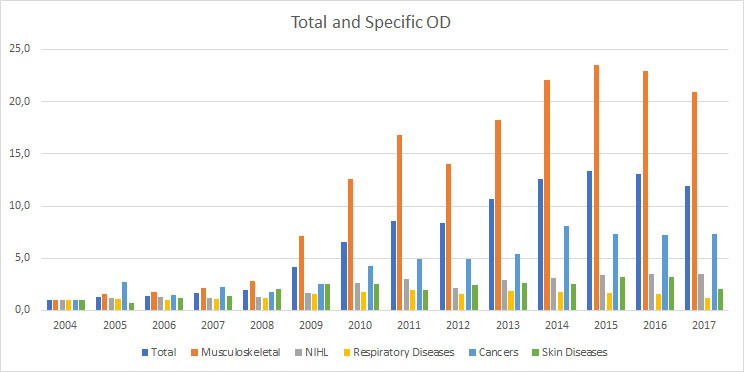
Incidence rate ratio of reported occupational diseases (ODs) in Italian agricultural sector during 2004–2017 with 2004 as reference year.

Over a 14-year period, increasing trends in incidence were found for MSDs (+26%; 95% CI 22% to 31%; see figure 1 and figure 2) and NIHL (+7%; 95% CI 1% to 12%). The annual incidence in occupational respiratory, cancer and skin diseases showed no change.

The eligibility criteria changed in 2008 showed an immediate effect for reporting occupational MSDs with an IRR of 2.9 (95% CI 2.65 to 3.14) and for NIHL with an IRR of 1.3 (95% CI 1.13 to 1.53). After 2008, MSDs showed a significant decreasing time trend with −9% (95% CI −6% to −12%). Respiratory diseases also showed an immediate reporting effect in 2008 with an IRR of 1.5 (95% CI 1.21 to 1.83).

## Discussion

The incidence of reported ODs in the last 10 years—with 74% (see figure 1) preliminary work-related MSDs—is high in the Italian agricultural sector. Worldwide, this predominance of work-related MSDs in agriculture has been reported in the literature.^7–9^ For ‘control’ ODs with no changed eligibility criteria in 2008, that is, occupational respiratory, dermal and cancer diseases, no statistically significant trend changes in OD reporting were found. Reported occupational respiratory diseases showed an increase in 2008; possibly the adoption of the new reporting rules also gave a small immediate impulse to the whole frame of reporting.

The stable trends, although the absolute numbers are relatively low, in occupational dermal and respiratory diseases in the Italian agricultural sector are in contrast to the declining trends in other Italian economic sectors^10^ and some other European countries.^10^

The data for ODs suffer some imprecision in diagnosis because they represent reported cases and not yet confirmed cases; however, they are relevant given the probably low selection bias of reported ODs to INAIL and that they show an increase of incidence of the diseases consistent with the updates and changes to the reporting rules (MSDs and NIHL in 2008). The trend of reporting did not change with regard to other ODs for which the criteria did not change. Moreover, after a relevant increase in OD reports in 2008–2016, reporting of MSDs showed a decreasing trend, suggesting the changes in criteria have brought about the reporting of already existing under-reported diseases and that reports now mainly concern ‘new’ post-2008 ODs. In this light, the increase of reporting observed is not indicative of a worsening working condition but mainly of an improved capacity around reporting. So changes and clarity in eligibility criteria seem to be reflected in changes in reporting, which is also shown in a Dutch study on the incidence rate of occupational low back pain-related ODs.^11^

In summary, 1.3% of workers in the Italian agricultural sector had an OD diagnosed and reported by a physician in 2017. Over a 14-year period, the annual incidence of reported ODs showed a statistically significant increase, mainly due to substantially increasing trend of MSDs and to a lesser extent of NIHL, 26% and 7%, respectively, and consistent with changed eligibility criteria for reporting ODs.
